# Supplementary material for: Translational contributions to tissue specificity in rhythmic and constitutive gene expression
Source: Genome Biol. 2017 Jun 16;18:116. doi: 10.1186/s13059-017-1222-2 (PMC5473967; doi:10.1186/s13059-017-1222-2)
Supplement: Supplementary file 7 — Expression plots for kidney and liver for the 178 common rhythmic genes of Fig. 3c. (ZIP 3338.28 kb) [file 13059_2017_1222_MOESM7_ESM.zip › set_D_shared(178)/Nampt_kidney_set_D.pdf]

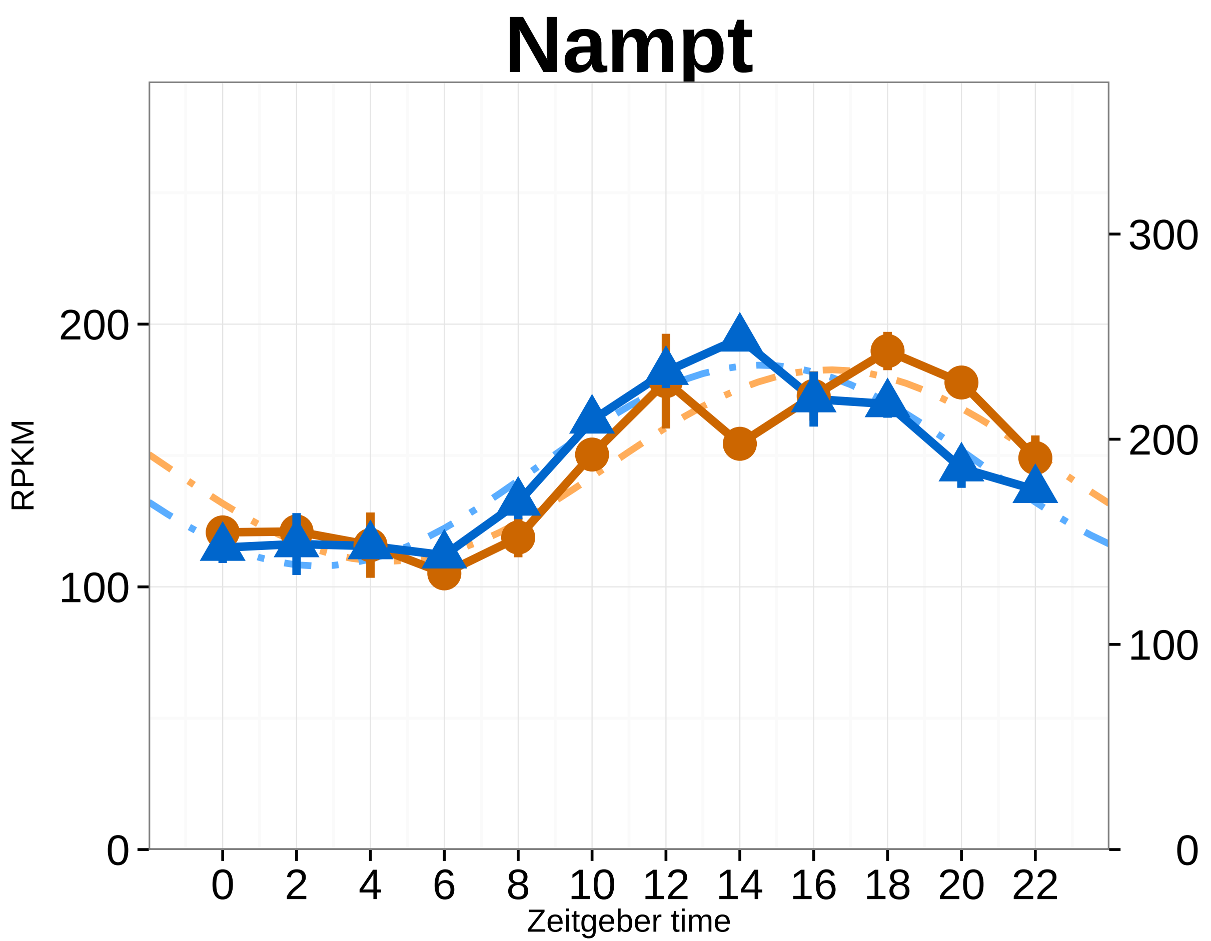
Nampt
RPKM
0
100
200
300
0 2 4 6 8 10 12 14 16 18 20 22
Zeitgeber time

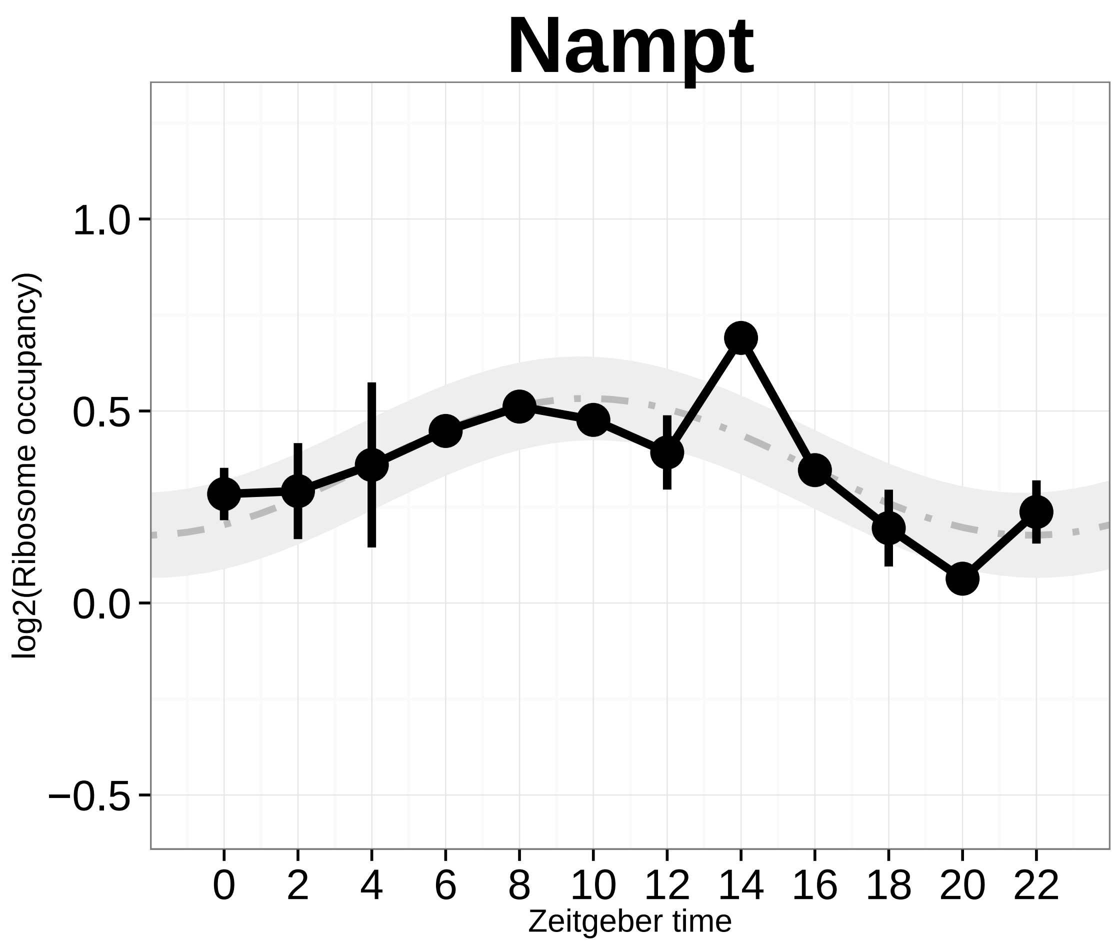
Nampt
log2(Ribosome occupancy)
−0.5
0.0
0.5
1.0
0 2 4 6 8 10 12 14 16 18 20 22
Zeitgeber time
